# Thyroid: Medullary Carcinoma

**DOI:** 10.4267/2042/48876

**Published:** 2013-04

**Authors:** Yash R. Somnay, David Schneider, Haggi Mazeh

**Affiliations:** Section of Endocrine Surgery, Department of Surgery, University of Wisconsin, K3/704 Clinical Science Center, 600 Highland Avenue, Madison, WI 53792, USA (YS, DS, HM)

## Abstract

Medullary thyroid cancers (MTC) are rare neuroendocrine tumors arising from the parafollicular C-cells of the thyroid. In this review, we provide a general overview of the classification, pathology, and clinical management of MTC. In the latter half, we survey the underlying genetic framework of MTC and its potential implications within a diagnostic and therapeutic context.

## Identity

### Other names

MTC

## Classification

### Note

Medullary thyroid cancers (MTC) are rare tumors of neuroendocrine origin that arise from parafollicular C-cells, which secrete a variety of peptides and hormones including calcitonin. As opposed to the more common papillary and follicular thyroid cancer subtypes, MTC represents a rare and under-characterized form of cancer, and may cause death if untreated (Taccaliti et al., 2011).

MTC can be either sporadic, usually isolated to one thyroid lobe, or familial, the latter of which is defined as part of the cancer syndrome known as Multiple Endocrine Neoplasia type 2 (MEN2) (Frank-Raue et al., 2010). MEN2 is the result of an autosomally dominant, missense, gain-of-function mutation in the RET (REarranged during Transfection) proto-oncogene. MEN2 can be further subclassified into MEN2A, MEN2B and Familial Medullary Thyroid Cancer (FMTC). MEN2A is defined by the occurrence of MTC in conjunction with pheochromocytomas and primary hyperparathyroidism. MEN2B is defined by the presence of MTC, pheochromocytomas, ganglioneuromatosis of the gastrointestinal tract, mucosal neuromas of the lips and tongue, and a marfanoid body habitus (Frank-Raue et al., 2010). FMTC occurs when MTC is the only clinical feature, and rarely with other endocrine neoplasias. Offspring of affected carriers of the RET mutation have a 50% chance of inheriting the mutation.

## Clinics and pathology

### Disease

Medullary thyroid cancer

#### Note

Patients with sporadic MTC usually present with a neck mass, while patients with hereditary MTCs who are diagnosed as mutation carriers should undergo prophylactic thyroidectomy before the onset of any symptoms. Sporadic MTC patients often present with metastases to cervical and paratracheal lymph nodes. The diagnosis of MTC is based on history, physical exam, calcitonin and CEA levels, imaging, and fine needle aspiration biopsy. Every patient with diagnosed MTC should undergo genetic evaluation for the presence of the RET mutation. Histologically, tumors appear containing hyperplastic parafollicular C-cells, and predominantly present bilaterally (Taccaliti et al., 2011). Sporadic MTC generally presents as a single tumor confined to one thyroid lobe.

The prognosis of MTC is better than the poorly-differentiated, malignant, anaplastic thyroid cancer, but is worse than the more well-differentiated and benign papillary and follicular thyroid cancers. Therefore, early diagnosis is necessary for improving recurrence and survival rates among these patients (Taccaliti et al., 2011).

### Phenotype / cell stem origin

MTC characteristically originates from neural crest cells. These cells arise from the convergence between the dorsal ectoderm and the neural tube. Neural crest cells eventually give rise to the chromaffin cells of the thyroid C-cells, in addition to the chief cells of the extra-adrenal paraganglia and adrenal medulla. Endocrine tumors that arise from thyroid C-cells during earlier stages of differentiation give rise to MTC. RET gene testing of germline DNA at the chromosomal region 10q11.2 must be performed in patients with a family history of MTC. This testing will identify hereditary MTC among 95% or more of individuals with MEN2A and MEN2B. Additionally, 88% of individuals with FMTC are identifiable through RET testing (National Cancer Institute, National Institutes of Health, www.cancer.gov).

### Etiology

Medullary thyroid cancer can be classified into 4 types:

1. Sporadic
2. Hereditary MEN2A
3. Hereditary MEN2B
4. Hereditary Familial Medullary Thyroid Cancer (FMTC)

### Epidemiology

In the United States, thyroid cancer comprises 3% of new malignancies occurring every year. Approximately 56,460 projected cases will be diagnosed annually, of which 1780 will result in death. MTC accounts for approximately 5-8% of all thyroid cancer. About 20-25% of MTC cases are the result of MEN2 syndromes. However, most reports of MTC are sporadic (National Cancer Institute, National Institutes of Health, www.cancer.gov). Among these, 56% occur as MEN2A, 9% as MEN2B, and 35% as FMTC (Frank-Raue et al., 2010).

MTC typically occurs in the third or fourth decade of life in MEN2A patients. MEN2B patients usually develop the syndrome in early childhood. The onset of disease in FMTC patients generally occurs during middle age.

### Clinics

Sporadic MTC generally presents as a single tumor confined to one thyroid lobe, while the familial form often presents bilaterally. Most MTC patients will present with a neck mass and may complain of hoarseness, dysphagia, and/or difficulty swallowing and breathing. MTC patients often present with metastases to cervical and paratracheal lymph nodes. Distant metastatic sites of MTC may include the lung, liver, and bones, and more rarely the brain and skin. Disseminated disease may cause symptoms of weight loss, lethargy, and bone pain. MTC patients often present with diarrhea due to the increased secretion of an intestinal electrolyte secondary to high plasma calcitonin levels. Flushing, similar to that present in carcinoid patients, often occurs as a result of the hypersecretion of calcitonin and other bioactive hormones.

### Pathology

Histologically, MTC tumors comprise hyperplastic parafollicular C-cells and predominantly present bilaterally in familial cases. MTC may be preceded by C-cell hyperplasia (CCH). However, CCH is a relatively common occurrence in unafflicted middle-aged adults (LiVolsi, 1997; Nose, 2011).

### Treatment

In sporadic MTC cases, total thyroidectomy and central lymph node dissection should be performed. Lateral lymph node dissection should be added when such involvement is identified. For patients who are known carriers of the RET mutation, prophylactic surgery should be offered. At present, guidelines recommend surgery at a certain age dictated by the type of mutation and its associated clinical course. Postponing surgical intervention until later in adulthood increases the likelihood of local recurrence and distant metastasis (Brandi et al., 2001).

Surgery for recurrent disease should be considered in the absence of distant metastases when remission is more achievable. If distant metastases are found, surgery may only be indicated if the patient presents with intractable symptoms. Such patients may also benefit from tumor debulking (Brandi et al., 2001).

For patients with metastatic MTC for which surgery cannot offer cure, there exist few chemotherapeutic options. Furthermore, MTC has been shown to respond poorly to radiotherapy-based regimens. However, some patients despite substantial metastatic burden can remain asymptomatic and live for many years (Taccaliti et al., 2011).

### Prognosis

The prognosis of MTC is worse than that of follicular and papillary thyroid cancer. Patients with hereditary MTC who undergo prophylactic surgery have an excellent prognosis (Raue, 1998). For all patients with MTC, 10-year survival rates vary between about 61% and 76% (Raue, 1998; Kebebew et al., 2000; Roman et al., 2006). MTC is often diagnosed using screens for calcitonin and carcinoembryonic antigen levels. Factors such as patient age, sex, calcitonin doubling-time, tumor volume and lymph node dissemination will dictate stage and prognosis.

## Genes involved and proteins

### RET (ret proto-oncogene)

#### Location

10q11.2

#### Note

The RET (REarranged during Transfection) gene is a member of the proto-oncogene cadherin, superfamily of Receptor Tyrosine Kinases that regulate processes affecting the growth and differentiation of neural crest cells, from which MTCs derive. When cytogenetically rearranged, RET can undergo oncogenic activation. Genetic diagnosis is crucial in order to differentiate familial from sporadic MTC and must be performed early on when a family history is remarkable (Frank-Raue et al., 2010).

### NKX2-1 (NK2 homeobox 1)

#### Location

14q13.3

#### Note

NKX2-1 encodes proteins involved in the budding and migration of the midline thyroid anlage. Its translated protein binds to the thyroglobulin promoter, which leads to the downstream expression of thyroid-specific genes and morphogenic processes. NKX2-1 is regarded as a thyroid-specific transcription factor. Mutations and deletions in this gene may be associated with sporadic MTC (Westerlund et al., 2008).

### BRAF (v-raf murine sarcoma viral oncogene homolog B1)

#### Location

7q34

#### Note

BRAF encodes a member of the Raf/Mil family of serine/threonine protein kinases and functions as a key regulator in the ERK signaling pathway. This pathway regulates cell division, differentiation, and bioactivity. BRAF gene mutations have been associated with sporadic MTC (Nikiforova et al., 2003).

### PTEN (phosphatase and tensin homolog)

#### Location

10q23.3

#### Note

PTEN encodes the phosphatidylinositol-3,4,5-trisphosphate 3-phosphatase protein, a known tumor-suppressor mutated in a variety of cancers. PTEN negatively regulates intracellular levels of phosphatidylinositol-3,4,5-trisphosphate in the AKT/PKB signaling pathway (Nose, 2011).

### HRAS (v-Ha-ras Harvey rat sarcoma viral oncogene homolog)

#### Location

11p15.5

#### Note

HRAS encodes an oncogene which is a member of the Ras family. These genes are related to the transforming genes of mammalian sarcoma retroviruses and behave as GTPase proteins. Therefore, HRAS mutations can lead to a variety of cancers including MTC. Studies have identified single nucleotide polymorphisms within HRAS among patient haplotypes shown to be associated with sporadic MTC. (Ruiz-Llorente et al., 2007; Barbieri, 2012).

### TP53 (tumor protein p53)

#### Location

17p13.1

#### Note

This gene encodes p53, which regulates such processes as apoptosis, cell-cycle arrest, and DNA repair. p53 binds DNA to induce expression of downstream genes that inhibit cell division, thus making it a tumor-suppressor. p53 mutants have been shown to bind poorly to DNA, thus repressing its tumor-suppressor activity. Regression analyses have linked TP53 genotype mutations to an inherited increase in the risk of sporadic MTC (Joao Bugalho et al., 2008; Barbieri, 2012).

### VEGFA (vascular endothelial growth factor A)

#### Location

6p12

#### Note

VEGFA encodes a growth factor of which mutations can cause proliferative and nonproliferative retinopathy in diabetic patients. Multiple isoforms have been identified due to upstream translation initiation sites of the AUG start codon. Furthermore, splice variants of different isoforms have also been identified, including ones either freely secreted or cell-associated. Studies have shown that VEGF expression in thyroid cancer correlates with tumor subtype and TNM stage. This may suggest that VEGF plays a role in the angiogenesis and metastasis of thyroid cancer (Ji et al., 2012).

### PTTG1 (pituitary tumor-transforming 1)

#### Location

5q35.1

#### Note

PTTG1 encodes a homolog of securin proteins, which function to block the separation of sister chromatids during anaphase until activation of the anaphase-promoting complex (APC), that it binds to upon APC activation. This gene is highly expressed in a variety of tumors and is mainly a cytosolic protein while partially localized in the nucleus. Levels of PTTG1 have been shown to correlate with MTC aggressiveness, among other cancers. Silencing PTTG1 has been shown to reduce MTC cell proliferation. This supports the hypothesis that PTTG1 may play an important role in MTC progression, thus serving as a therapeutic target (Zatelli et al., 2010).

### ESR2 (estrogen receptor 2 (ER beta))

#### Location

14q23.2

#### Note

ESR2 encodes an estrogen receptor consisting of a nuclear receptor transcription factor containing a DNA-binding domain on its N-terminus. When 17-beta-estradiol binds to ESR2, the complex forms either a homodimer or heterodimer with ESR1. In normal physiology, ESR1 plays a role in sexual development, reproduction, as well as bone and tissue development, but may be mutated in a variety of cancers.

ESR2, but not ESR1, is present in thyroid tissue, but there exist no notable associations between ESR2 expression and MTC (Vaiman et al., 2010).

### NRAS (neuroblastoma RAS viral (v-ras) oncogene homolog)

#### Location

1p13.2

#### Note

The N-ras oncogene encodes a membrane protein that shuttles between the Golgi apparatus and the plasma membrane. This shuttling is regulated through palmitoylation and depalmitoylation by the ZDHHC9-GOLGA7 complex. The encoded protein, which has intrinsic GTPase activity, is activated by a guanine nucleotide-exchange factor and inactivated by a GTPase-activating protein. Mutations in this gene have been associated with somatic rectal cancer, follicular thyroid cancer, autoimmune lymphoproliferative syndrome, Noonan syndrome, and juvenile myelomonocytic leukemia (Schulten et al., 2011; Almeida and Hoff, 2012).

### EGFR (epidermal growth factor receptor)

#### Location

7p12

#### Note

EGFR encodes a transmembrane glycoprotein receptor with kinase activity and is a member of the epidermal growth factor family. Binding of the epidermal growth factor to the EGFR induces receptor dimerization and tyrosine autophosphorylation, in turn causing cell growth and proliferation. EGFR gene mutations have been shown to cause lung cancer. Alternatively spliced transcript variants encoding different isoforms have been described. Targeting EGFR through small-molecule inhibitors has been shown to be useful in treating various cancers including MTC. Recently, vandetanib (ZD6474), an EGFR inhibitor, was approved for treating progressive and symptomatic MTC (Almeida and Hoff, 2012).

### NFKB1 (nuclear factor of kappa light polypeptide gene enhancer in B-cells 1)

#### Location

4q24

#### Note

This 105 kD protein may undergo 26S proteasome processing to produce a 50 kD protein, which is the DNA-binding subunit of the NF-kappa-B (NFKB) protein complex. NFKB serves as a transcriptional regulator activated by various cell stressors including cytokines, free radicals, UV radiation, and bacterial or viral products. Upon activation, NFKB enters the nucleus where it induces gene expression in a variety of cell-survival and immune-related functions. Super-activation of NFKB has been linked to numerous inflammatory diseases as well as perturbations in cell growth and development. Recently, NFKB has been shown to play an important role in thyroid cancer cell proliferation and regulation of anti-apoptotic mechanisms inherent to the cancer phenotype (Gallel et al., 2008; Pacifico and Leonardi, 2010).

### STAT3 (signal transducer and activator of transcription 3 (acute-phase response factor))

#### Location

17q21.31

#### Note

The STAT3 gene encodes an isoform of the STAT protein family. These proteins are phosphorylated by receptor-associated kinases in response to growth factors and cytokine stimuli. STAT3 then translocates to the nucleus as a complex, in order to activate the transcription of downstream genes involved in cell growth and apoptosis. Three alternatively spliced transcript variants producing different isoforms have been identified. Recent studies have demonstrated that FMTC-RET mutants activate the Ras/ERK1/2 pathway upstream of the STAT3 Ser^727^ pathway. STAT3 may thus play an important role in the oncogenic transformation of thyroid cancer (Plaza-Menacho et al., 2007).

### MMP2 (matrix metallopeptidase 2 (gelatinase A, 72kDa gelatinase, 72kDa type IV collagenase))

#### Location

16q13-q21

#### Note

Matrix metalloproteinases (MMP) are involved in the disintegration of extracellular matrices during normal physiologic processes such as reproduction, tissue remodeling, embryonic development and wound healing, but also in cancer metastasis. MMP2 degrades type IV collagen which plays a structural role in basement membranes. Two transcript variants encoding different isoforms have been found for this gene. A recent study assessing a panel of MTC cancer specimens found that expression of MMP2 could be used as a prognostic tool (Cavalheiro et al., 2008).

### NOTCH1 (notch 1)

#### Location

9q34.3

#### Note

Notch1 is a member of the Notch transmembrane protein family (Notch1-4) which possesses an extracellular domain of epidermal growth factor-like repeats and an intracellular domain containing different domain types. Notch signaling is initiated intercellularly following physical interaction between ligands (delta serrate) on adjacent cells and is evolutionarily conserved. This protein is cleaved in the trans-Golgi network and presented on the cell surface as a heterodimer. Notch1 has been identified as a tumor-suppressor in MTC, in addition to other neuroendocrine tumors such as carcinoids. In MTC, Notch1 is expressed at very low to absent levels; however, upregulating NOTCH1 expression reduces MTC cell proliferation and phenotypic expression (Kunnimalaiyaan et al., 2006).

### GFRA1 (GDNF family receptor alpha 1)

#### Location

10q26.11

#### Note

Glial cell line-derived neurotrophic factor (GDNF) is a glycosylphosphatidylinositol-linked receptor on the cell surface and plays key roles in the differentiation and survival of neurons. It is also involved in the regulation of RET tyrosine kinase activity. Multiple, alternatively spliced transcript variants have been described for various GFRA1 isoforms. Furthermore, germline polymorphisms in RET and GFRA1, as well as correlations with genetic predispositions to developing sporadic MTC have been described. Modulating these polymorphisms has been thought to affect clinical features of the disease as well. (Severskaia et al., 2006).

### KRAS (v-Ki-ras2 Kirsten rat sarcoma viral oncogene homolog)

#### Location

12p12.1

#### Note

Kirsten RAS oncogene homolog is a small GTPase and member of the mammalian RAS gene family. Mutations can be caused by an amino acid substitution, leading to oncogenic activation in various malignancies. Alternative splicing variants of two isoforms have been described. Mutation screening of KRAS may be warranted, but is still inconclusive (Schulten et al., 2011).

### MTOR (mechanistic target of rapamycin (serine/threonine kinase))

#### Location

1p36.2

#### Note

MTOR serves as a target of the FKBP12-rapamycin complex, which potentiates immunosuppression and cell-cycle inhibition. It belongs to a family of phosphatidylinositol kinase-related kinases which regulate cell processes such as growth and survival in response to DNA damage, free radical damage and nutrient deprivation. MTOR signaling is aberrantly activated in MTC, especially in tissues harboring germline RET mutations (Rapa et al., 2011).

## Figures and Tables

**Figure 1:**
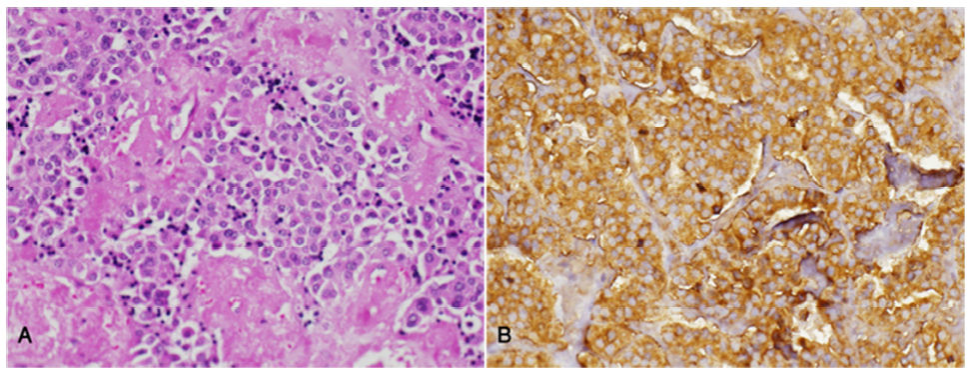
**A.** Medullary thyroid carcinoma featuring groups of cells with polygonal to elongated cytoplasms and round-to-oval nuclei with indistinct nucleoli. Note the amyloid deposition in the stroma (H&E, x200). **B.** Strong immunopositivity for calcitonin in all tumor cells (immunoperoxidase staining, x200).
